# Redistribution of critical drugs in shortage during the first wave of COVID-19 in France: from operating theaters to intensive care units

**DOI:** 10.1186/s40545-022-00425-z

**Published:** 2022-04-01

**Authors:** Claire Chapuis, Rémy Collomp, Laura Albaladejo, Hugo Terrisse, Stéphane Honoré, Jean-Luc Bosson, Pierrick Bedouch, Pierre Albaladejo

**Affiliations:** 1 Pôle Pharmacie, Pôle Anesthésie-Réanimation, CHU Grenoble Alpes, CS10217, 380143 Grenoble cedex 9, France; 2 Pôle Pharmacie Stérilisation, CHU De Nice, Société Française de Pharmacie Clinique (SFPC), Nice, France; 3 Equipe Themas, Timc-Imag Umr5525, Université Grenoble Alpes, Grenoble, France; 4 Pôle Pharmacie, Hôpital De La Timone, Société Française de Pharmacie Clinique (SFPC), Marseille, France; 5 Pôle Santé Publique, CHU Grenoble Alpes, Grenoble, France; 6 Pôle Pharmacie, CHU Grenoble Alpes, Société Française de Pharmacie Clinique (SFPC), Grenoble, France; 7 Pôle Anesthésie-Réanimation, CHU Grenoble Alpes, Société Française d’Anesthésie Réanimation (SFAR), Grenoble, France

**Keywords:** (MeSH): anesthesia, Critical care, Anesthetics, Shortage, Survey

## Abstract

**Background:**

Tension in the supply of highly consumed drugs for patients with COVID-19 (propofol, midazolam, curares) led the French government to set up a centralized supply of hospitals with distribution based on the number of resuscitation beds in March 2020. The French Societies of Clinical Pharmacy and of Anesthesia and Critical Care aimed to evaluate the changes in total needs and the distribution between anesthesia and critical care activities (CCU), to prepare resumed surgical activity.

**Methods:**

National declarative survey among pharmacists, via an online form (SurveyMonkey®), was conducted in April and May 2020. The analysis focused on quantities dispensed during the whole year 2019, and March and April of year 2019 and 2020 for the drugs subject to quota, and on their distribution in CCU and operating theaters.

**Results:**

For the 358 establishments (47% public, 53% private), dispensations in CCU in March 2020 compared to March 2019 increased, respectively: propofol (+81%), midazolam (+125%), cisatracurium (+311%), atracurium (+138%), rocuronium (+119%); and decreased for anaesthesia: propofol (−27%), midazolam (-10%), cisatracurium (−19%), atracurium (−27%), rocuronium (+16%).

**Conclusions:**

Variation of dispensations between CCU and others was directly related to the increase of COVID patients in CCU and the decrease in surgical activity. Each establishment could receive up to five or six different presentations and concentrations, leading to a major risk of medication error. This collaborative national survey provided accurate data on the drugs’ usual consumption. This work emphasized the need for a strong collaboration between pharmacists and anesthesiologists and intensive care physicians. It was further used by the Health Ministry to adjust the drug distribution.

## Background

During the first wave of the COVID-19 pandemic, the maximal alert in French hospitals was announced on March 13th 2020. Consequently, non-urgent surgical activity was postponed or cancelled. Priority was given to Intensive Care Units (ICU) for fear of running out of essential drugs for critical care patients. National health agencies have been closely monitoring the medical product supply chain with the expectation that it may be impacted by the COVID-19 outbreak, potentially leading to supply disruptions or shortages of drug products worldwide. From March 2020, we experienced the supply tensions in highly consumed medicines for the care of patients with COVID, so a new supply system was set up by the French Ministry of Health with a controlled distribution of these medicines between hospital establishments at national level, with a possible regional adjustment via Health Regional Agencies. Targeted drugs were sedatives and neuromuscular blocking agents, massively used to manage severe COVID-19 pneumonia but also essential for anesthesia practice, namely: propofol, midazolam, cisatracurium, atracurium and rocuronium. The distribution key was then essentially based on the number of resuscitation beds (COVID and non-COVID), without initially taking into account the residual activity of anesthesia for urgent intervention. To also integrate the anesthesia component, which already existed at a small volume but which would regain importance with future gradual resumptions of surgical activities, the French Society of Clinical Pharmacy (SFPC) and the French Society of Anesthesia and Critical Care (SFAR) aimed to assess and proposed taking into account the ratio of these drugs between anesthesia activities (operating theaters-OT, and post-anesthesia care units-PACU) and critical care activities (intensive care units-ICU, etc.) for each establishment (private or public, with or without resuscitation activities) during the first COVID-19 wave in France.

## Methods

Pharmacists of public (University Hospital Centers and non-University Hospital Centers) and private hospitals, with or without ICU beds, were asked to complete the survey at two times: first between April 27 and 30, 2020 and then between May 11 and May 29, 2020. They completed the data concerning the periods: March and April 2019, the whole year 2019, March and April 2020.

The status of stocks of targeted drugs (propofol, midazolam, atracurium, cisatracurium, rocuronium) was already registered in the "MaPUI" software (MaPUI Labs, Cesson Sévigné, France), created for this purpose by a French start-up. At the time of the first stage of the survey, the hospitals without a resuscitation unit were not yet included in the "MaPUI.fr" system.

The dispensing volumes of the 5 quota drugs from the Pharmacy to: (1) ICU (2) OT and PACU), (3): other services (home hospitalization, palliative care, etc.…) (for midazolam only), were collected and analyzed by comparing the periods March 2020 to March 2019 and April 2020 to April 2019 (with the full year 2019 as a reference), to:

- Assess overall quantitative changes (total needs)
- Evaluate the distribution proportions between OT-PACU/ICU/other services to identify the respective distribution of drugs.
- Have useful data for the prospective preparation for resumption of activity (better estimate of needs).

### Data management

Survey was carried out on SurveyMonkey® software (San Mateo, California, USA) via an online form. The invitation to participate was sent through SFAR and SFPC networks, the Conference of Pharmacists of University Hospitals and professional unions.

The cleaning of the database was carried out as follows:

- Search for duplicates by two observers on two different inputs (making sure that they are not different establishments within the same structure). The data retained were those that were the most complete or the most recent.
- Exclusion of establishments that had not provided any quantitative data for the drugs evaluated.
- Research and exclusion of establishments for which the extreme quantitative data were not consistent between March and April 2019 and the year 2019 (search for outliers: analysis of the extremes with a search for the maximum and comparison with other establishments (of the same type), consistency between extremes, medians, quartiles and means). In this study, we used the mean. Indeed, we can infer the total quantity dispensed by multiplying the mean by the number of hospitals, which cannot be done using the median.
- The missing data were replaced by zero for the quantitative data, considering that they had been completed in full.

For the establishments, which provided data for critical care units in March 2019, two analyzes were carried out for March and April 2019, March and April 2020, and the year 2019:

- The average quantity (in grams) dispensed by type of activity (critical care, operating theaters–interventional units and PACU, and others).
- The respective distribution and variations of dispensations for critical care, operating theaters–interventional units and PACU, and other services. An increase in the quantities dispensed resulted in a positive variation and a decrease resulted in a negative variation.

This study did not concern patients, therefore, was not subject to the need for medical ethical approval, and no consent was needed (according to French recommendations)*.*

## Results

Between April 27 and 30, 2020 and between May 11 and May 29, 392 and 188 establishments responded to the survey for March and April, respectively. After cleaning the database, data analysis was carried out on 358 establishments for March and 164 establishments for April. Annex 1 presents the breakdown by type of establishment and by region. The most affected regions in France were around Paris and East of France.

**Fig. 1:**
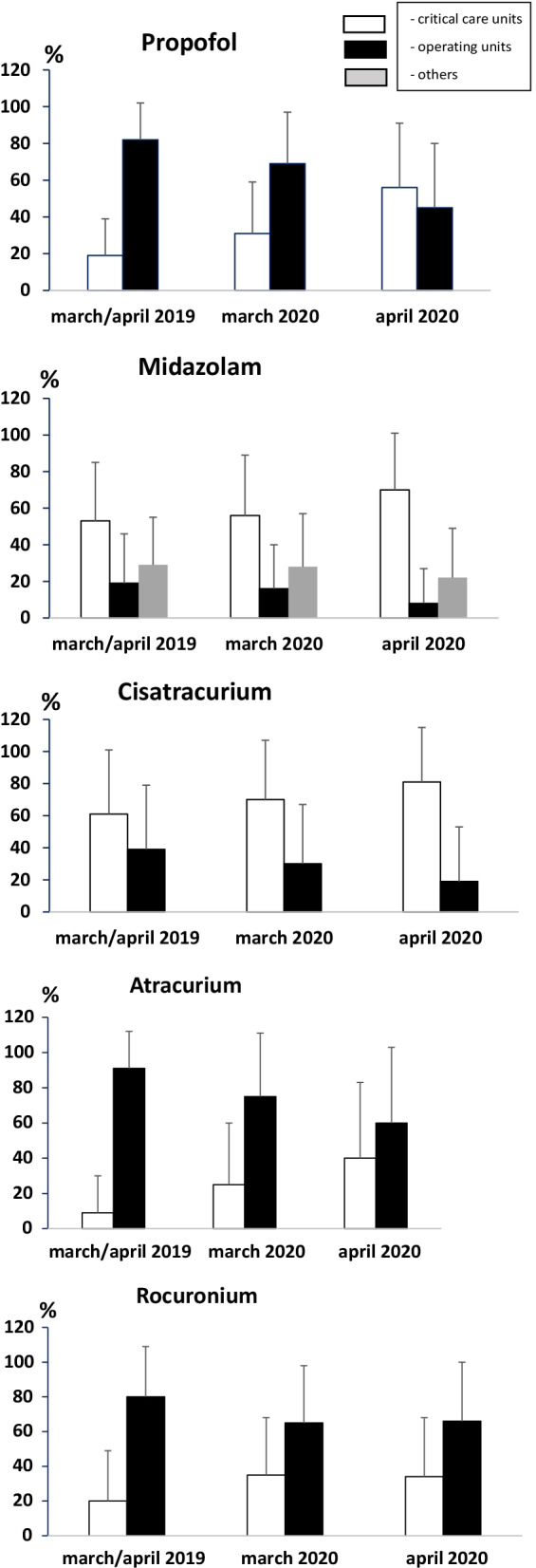
Distribution of drug dispensations in hospitals with critical care units (mean, standard deviation)

Quantities in grams of the five drugs dispensed by establishment and type of activity for centers with critical care activity are provided in Table 1. Figure 1 shows the distribution of drug dispensations in hospitals with critical care units.

**Table 1:** Quantities (in grams) dispensed by establishment and type of activity (centers with critical care activity; in March *n* = 237, in April *n* = 118)

Mean (± 1 standard deviation) (gram)	Critical care	Operating theaters	Others	Total
Year 2019				
Propofol	1847 ± 4001	5311 ± 6269	–	7158 ± 9590
Midazolam	151 ± 319	15 ± 26	48 ± 72	214 ± 375
Cisatracurium	79 ± 170	28 ± 60	–	106 ± 220
Atracurium	30 ± 108	158 ± 212	–	189 ± 275
Rocuronium	7.5 ± 20	43 ± 101	–	50 ± 111
March 2019				
Propofol	170 ± 365	457 ± 526	–	627 ± 822
Midazolam	14 ± 31	1.3 ± 2.4	4.2 ± 6.1	19 ± 36
Cisatracurium	6.9 ± 15	2.8 ± 6.5	–	9.7 ± 19
Atracurium	2.9 ± 11	14 ± 19	–	17 ± 25
Rocuronium	0.7 ± 1.9	3.3 ± 7.2	–	4.0 ± 8.2
March 2020				
Propofol	322 ± 627	358 ± 451	–	680 ± 992
Midazolam	33 ± 54	2.4 ± 6.3	6.3 ± 9.7	41 ± 63
Cisatracuriu m	29 ± 55	2.8 ± 7.4	–	32 ± 60
Atracurium	15 ± 49	12 ± 22	–	28 ± 59
Rocuronium	1.9 ± 4.8	3.9 ± 8.6	–	5.9 ± 11
April 2019				
Propofol	214 ± 425	514 ± 616		727 ± 981
Midazolam	21 ± 39	2 ± 4.7	5 ± 7.3	28 ± 46
Cisatracurium	10 ± 18	3.1 ± 6.6		13 ± 24
Atracurium	3 ± 14	15 ± 21		18 ± 30
Rocuronium	0.8 ± 1.7	5.2 ± 12		6.1 ± 12
April 2020				
Propofol	537 ± 833	177 ± 245		714 ± 993
Midazolam	59 ± 89	1.7 ± 3.5	19 ± 144	80 ± 189
Cisatracurium	44 ± 75	2.1 ± 5.7		46 ± 78
Atracurium	43 ± 132	7.7 ± 20		50 ± 146
Rocuronium	5.5 ± 26	5.3 ± 10		11 ± 31

The variation of dispensations March 2019/March 2020 for critical care units was, respectively: propofol (+81%), midazolam (+125%), cisatracurium (+311%), atracurium (+138%), rocuronium (+119%); for anesthesia: propofol (−27%), midazolam (−9.5%), cisatracurium (−19%), atracurium (−27%), rocuronium (+16%).

The variation of dispensations April 2019 / April 2020 for critical care units further evolved and resulted in an increase, respectively: propofol (+165%), midazolam (+204%), cisatracurium (+302%), atracurium (+350%), rocuronium (+43%). For anesthesia, it resulted in a decrease: propofol (−70%), midazolam (−67%), cisatracurium (−55%), atracurium (−65%), rocuronium (0%).

A shortage of at least one of the five drugs concerned by the survey was reported by 28% of establishments. Nineteen percent of establishments have received at least one specialty with a foreign labelling (not French).

## Discussion

This large panel corresponding to this declarative survey was representative of the types of establishments and regions. The months of March and April 2019 chosen as a comparison of the months of March and April 2020 (beginning of the epidemic and peak of the epidemic in many regions, and forecast increase in allocations in the affected establishments a little later) appeared to be representative of the full year 2019.

The reduced quantities observed overall in March 2020 for operating theaters, interventional units and PACU are related to the decrease in anesthesia activity linked to surgical activity, which was strongly reduced during this period. In parallel, we could see the significant increase in dispensations of the five drugs studied in critical care in connection with the treatment of COVID patients. The order of magnitude of this variation goes from quantity doubled for propofol and midazolam, and quadrupled for curares. This survey shows that the large increase in anesthesia drug consumption in critical care, appearing over a very short period and in a homogeneous manner across all establishments, was greater than the decrease observed on the surgical side. This observation was of utmost importance to calibrate distribution of stocks among hospitals between ICU and OT and to mitigate the impact of drug shortage on anesthesia activities. Indeed, there was no data at that time on the residual OR activities related to urgent surgical interventions.

For the month of March 2020, among curares, cisatracurium was primarily reserved for resuscitation, indeed it is the only curare with an authorization for continuous infusion in ICU. Prescriptions were reported on atracurium in anesthesia. We then observed the use of all curares in resuscitation to save or replace cisatracurium (without information on the usual practices of each establishment). We observed the same trend, increased distribution in intensive care, for propofol and midazolam but at a lower level. This difference in effect was probably due to co-administration of other sedative drugs to save propofol and/or midazolam (neuroleptics, benzodiazepine other than midazolam, alpha 2 agonists). Other services such as home hospitalization and palliative care have not been totally devoid of midazolam (the decrease can be explained by the saving strategies, which were implemented).

A shortage of at least one of the five drugs involved in the study was reported by 28% of establishments. Nineteen percent of establishments received at least one foreign specialty. The management of drug risk appeared critical, by the fact that the presentations received following the quota distribution by the government were not necessarily those of the therapeutic formulary of the hospital, each establishment being able to receive up to five or six different presentations and different concentrations, leading to a major risk of medication error. The distribution method could have been improved on this aspect, to deliver only or in the majority, the preexisting specialties in the establishment. It should be noted that other SFPC/SFAR collaborative work was also published [1], to propose recommendations aimed at globally preventing drug risk during this COVID period.

Many pharmacists worldwide shared their experience about COVID-19 pharmaceutical responses, emphasizing their roles in the intensive care units and the close collaboration with physicians and nurses [2–18]. Indeed, facing the shortage of drugs, use of unusual drugs with many interactions and resulting therapeutic risks, pharmacists were at the frontline and profoundly involved in securing medication use. The ongoing supply shortage of curares has led pharmacists in some hospitals (e.g., Lille, France) to produce curares, such as cisatracurium.

This situation also raise the question of securing critical drugs at a national or international level. It is noteworthy that even in the prepandemic period these drugs were regularly in shortage.

## Conclusions

The data collected during this national survey covering a large panel of establishments, of different size and geographic location, provided precise information on the distribution of consumption of quota drugs between intensive care and operating theaters, and other services (home hospitalization and palliative care) over the period of March and April 2020 (corresponding to the start and the peak of the first wave of COVID-19 pandemic in France), as well as a first estimate of needs for the period of resumption of activity via data of the global year 2019.

This first collaborative national survey made it possible to analyze concretely the increase in the consumption of drugs under tension and to know their usual operating consumption. This reinforced the essential collaboration between pharmacists, intensive care physicians and anesthesiologists. It helped the authorities to better adjust the drug distribution.

## Data Availability

Upon request.

## Annex 1: Types of Hospitals by region of France

**Table:**

*n*	Public non university hospital		Public university hospitals		Private hospitals		Total	
	March 2020	April 2020	March 2020	April 2020	March 2020	April 2020	March 2020	April 2020
Auvergne-Rhône-Alpes	31	19	3	3	26	9	60	31
Bourgogne-Franche-Comté	6	3	3	2	11	2	20	7
Bretagne	7	1	3	2	12	5	22	8
Centre-Val de Loire	9	6	2	1	2	1	13	8
Corse	–	0	–	0	1	0	1	0
France outre-mer	1	0	4	1	3	1	8	2
Grand Est	11	5	1	2	11	5	23	12
Hauts-de-France	12	9	3	2	12	7	27	18
Île-de-France	9	10	5	1	35	13	49	24
Normandie	14	17	–	2	16	7	30	26
Nouvelle-Aquitaine	16	1	3	3	25	3	44	7
Occitanie	14	5	3	1	21	5	38	11
Pays de la Loire	6	14	2	3	13	5	21	22
Provence-Alpes-Côte d'Azur	13	5	3	2	20	5	36	12
Total	149 (38%)	95 (50%)	35 (9%)	25 (13%)	208 (53%)	68 (36%)	392	188
